# Dose–Volume Constraints fOr oRganS At risk In Radiotherapy (CORSAIR): An “All-in-One” Multicenter–Multidisciplinary Practical Summary

**DOI:** 10.3390/curroncol29100552

**Published:** 2022-09-27

**Authors:** Silvia Bisello, Savino Cilla, Anna Benini, Raffaele Cardano, Nam P. Nguyen, Francesco Deodato, Gabriella Macchia, Milly Buwenge, Silvia Cammelli, Tigeneh Wondemagegnehu, A. F. M. Kamal Uddin, Stefania Rizzo, Alberto Bazzocchi, Lidia Strigari, Alessio G. Morganti

**Affiliations:** 1 Department of Experimental, Diagnostic and Specialty Medicine-DIMES, Alma Mater Studiorum University of Bologna, 40128 Bologna, Italy; 2 Radiation Oncology, IRCCS Azienda Ospedaliero-Universitaria di Bologna, 40128 Bologna, Italy; 3 Medical Physics Unit, Gemelli Molise Hospital-Università Cattolica del Sacro Cuore, 86100 Campobasso, Italy; 4 Department of Radiation Oncology, Howard University College of Medicine, Washington, DC 20059, USA; 5 Radiation Oncology Unit, Gemelli Molise Hospital-Università Cattolica del Sacro Cuore, 86100 Campobasso, Italy; 6 Department of Radiation Oncology, Addis Ababa University, Addis Ababa 1000, Ethiopia; 7 Department of Radiation Oncology, United Hospital Limited, Dhaka 1205, Bangladesh; 8 Service of Radiology, Imaging Institute of Southern Switzerland, Ente Ospedaliero Cantonale (EOC), 6500 Lugano, Switzerland; 9 Diagnostic and Interventional Radiology, IRCCS Istituto Ortopedico Rizzoli, 40136 Bologna, Italy; 10 Department of Medical Physics, IRCCS Azienda Ospedaliero-Universitaria di Bologna, 40128 Bologna, Italy

**Keywords:** literature review, radiotherapy, organs at risk, dose–volume constraints, guideline

## Abstract

Background: The safe use of radiotherapy (RT) requires compliance with dose/volume constraints (DVCs) for organs at risk (OaRs). However, the available recommendations are sometimes conflicting and scattered across a number of different documents. Therefore, the aim of this work is to provide, in a single document, practical indications on DVCs for OaRs in external beam RT available in the literature. Material and Methods: A multidisciplinary team collected bibliographic information on the anatomical definition of OaRs, on the imaging methods needed for their definition, and on DVCs in general and in specific settings (curative RT of Hodgkin’s lymphomas, postoperative RT of breast tumors, curative RT of pediatric cancers, stereotactic ablative RT of ventricular arrythmia). The information provided in terms of DVCs was graded based on levels of evidence. Results: Over 650 papers/documents/websites were examined. The search results, together with the levels of evidence, are presented in tabular form. Conclusions: A working tool, based on collected guidelines on DVCs in different settings, is provided to help in daily clinical practice of RT departments. This could be a first step for further optimizations.

## 1. Introduction

Radiation therapy (RT) is an effective cancer treatment. However, like any other therapy, RT is associated with the risk of side effects. In particular, RT can produce both early (acute) and delayed (late) damage to organs at risk (OaRs). Therefore, since the early applications of RT, interest has grown in ways to reduce radiation-induced toxicity.

In particular, starting from the 1970s, progressively more detailed indications on safe dose limits became available in the literature. In fact, after the pioneering work of Rubin and Cassaret [1] and the historical so-called Emami’s paper [2], the Quantitative Analyses of Normal Tissue Effects in the Clinic (QUANTEC) guidelines [3,4,5], based on dose/volume constraints (DVCs), were published in 2010. Subsequently, with the growing interest in hypofractionated treatments, especially delivered with stereotactic techniques, several recommendations were published on DVCs to be used with this dose fractionation [6,7,8,9,10]. In addition, specific DVC guidelines were published in particular clinical settings, such as Hodgkin’s lymphomas [11], breast carcinomas [12,13,14], and pediatric cancers [15,16,17,18,19,20]. Finally, in the last few years, in parallel with the introduction of stereotactic ablative RT of ventricular arrythmia (STAR) [21,22,23], specific DVCs for this treatment were also proposed [6,8,21,22,24]. Therefore, a large number of guidelines or recommendations is now available to guide RT prescription and treatment plan evaluation and comparison.

Unfortunately, this information is contained in a plethora of sometimes conflicting publications. Therefore, a quick consultation to find clear and unambiguous indications is not always easy. Therefore, the aim of this work is to present, in a single document, practical indications on DVCs for different clinical settings and dose fractionations.

## 2. Materials and Methods

For the purposes of this project (CORSAIR: dose–volume constraints for organs at risk in radiotherapy), a multidisciplinary working group was established, including radiation oncologists, medical physicists, and radiologists. Colleagues from other Italian, American, African, and Asian centers were added to a first original nucleus made up of staff from our center (Bologna University). In particular, the contribution of some colleagues from developing countries was requested in order to verify the possibility of clearly and correctly interpreting the recommendations also in low-medium-resourced settings.

A literature search was performed in March 2022, using PubMed and without time limits, with different combinations of the following keywords: “dose/volume”, “constraints”, “organs at risk”, and “radiotherapy”. Only papers in English were considered. Moreover, the bibliographic list of one hundred and twenty publications was screened in order to identify other relevant sources. Furthermore, for convenience, only recommendations based on simple indications of dose and volume limit values (or percentages) were included in this collection. In addition, we consulted the National Comprehensive Cancer Network (NCCN) guidelines in all cases in which, in the indication of radiotherapy, the DCVs of specific OaRs were presented and precisely in the case of lymphomas [11], lung cancers [25] and tumors of the esophagus [26], stomach [27], and anus [28]. The Global Quality Assurance of Radiation Therapy Clinical Trials Harmonization Group (GHG) contouring guidelines were used as the reference list and nomenclature system of the OaRs [29]. Furthermore, the OaR anatomical descriptions and landmarks were extracted from the same document and shown with the DVCs in tabular form. The anatomical descriptions were classified by level of reliability in their use for DVC evaluation. The classification was performed as follows: α: international guidelines or expert consensus in RT contouring, β: validated anatomical description for RT contouring from single institution, γ: anatomical or radiological descriptions from dedicated books or papers, δ: anatomical definition for RT contouring used in planning studies.

The results recorded during this research were independently verified by three authors from different centers and then summarized in tabular form. In the tables we referred to a different modality of fractionation and in particular to conventional fractionation, moderate hypofractionation, and ultra-hypofractionation. However, it should be considered that the definitions of fractionation refer to the dose per fraction administered to the tumor, which is generally different from that to the OaRs. Therefore, particular caution is required in the use of information contained in the tables, as well as the radiobiological knowledge on the impact of the different fractions and the clinical experience in this topic. In particular, six different tables related to different RT treatment settings were drawn up. In addition, together with the recommended DVC values, we reported the grade of recommendation (for example: mandatory, recommended, optimal, or acceptable) if included in the reference publication. Moreover, the optimal imaging technique for delineating the specific OaR was included in the tables if included in the selected publications. In addition, in order to provide users with a critical assessment of the DVCs, we categorized the source of recommendation as follows: (A) international guidelines; (B) literature reviews on clinical or planning studies; (C) data from the results of clinical or planning studies; (D) expert opinions or DVCs used in prospective trials. Moreover, when different sources presented different values of the same DVC, we included in the tables only the one with the highest level of evidence. Therefore, we included recommendations with “B-D” source of recommendation only in case of lack of level “A” DVCs.

Finally, common abbreviations in the literature were used in the tables: V = volume receiving a dose ≥ Gy, D = dose received by % of the organ volume, D = dose received by γ cm^3^ (the cubic centimeters) of the organ volume, DMAX = maximum dose received by the organ, DMEAN = mean dose received by the organ. Volumes and doses were expressed as percentage (%) or absolute values (cm^3^ or Gy, respectively).

## 3. Results

Six hundred and seventy-five papers/documents/websites were examined. The results of our search are shown in Table 1 (DVCs for all treatments), Table 2 (anatomical description of organs at risk). Supplementary Table S1 (DVCs for emerging OaRs), Supplementary Table S2 (DVCs for RT of Hodgkin’s lymphoma), Supplementary Table S3 (DVCs for RT of breast cancers), Supplementary Table S4 (DVCs for RT of pediatric tumors), and Supplementary Table S5 (DVCs for stereotactic ablative RT of ventricular arrythmia) are available as Supplementary Material.

## 4. Discussion

The initial aim of this work was to produce practical guidelines within our institution and, thus, to provide radiation oncologists with a quick and user-friendly synopsis without (or with little) ambiguity. We subsequently decided to share this work and to involve a multidisciplinary and international team of authors to write this paper.

In drafting these guidelines, we avoided entering different values (from different sources) for the same DVC. For this reason, we identified criteria for selecting the data to be included. Therefore, we scored the source of recommendation as previously reported. 

Obviously, this choice reduced the amount of the presented information but we hope that the tables will be used as an index to the literature where further relevant details can easily be found. Furthermore, grading the evidence provides the reader with an estimate of the single recommendation relevance.

Obviously, the use of these recommendations cannot be “automatic” but necessarily requires management by operators with knowledge and experience on the details needed for an informed and expert use of dose/volume constraints. For example, the evaluation of dose/volume histograms for OaRs must take into account various aspects, such as patient age and comorbidities, spatial characteristics of the radiotherapy dose distribution, treatment aims, and any symptoms that are manageable or not with other treatments.

This collection of DVCs for OaRs has obvious limitations. Not all available information was included, only some has been selected. To this end we used well-defined objective criteria which, however, were inevitably the result of a subjective and somehow arbitrary choice for the authors. Therefore, we are aware that different criteria would have led to recommendations different from those presented here. In addition, many DVCs came from publications, such as the QUANTEC guidelines, based on the results of clinical studies where RT was mainly delivered with the 3D technique. However, it is known that different and new techniques may require different and new DVCs. For example, in the Allen et al. experience, where the DVC V_20 Gy_ was met, 6 out of 13 patients with pleural mesothelioma undergoing intensity-modulated RT died from radiation-induced pneumonitis. From the analysis of their data, the authors concluded that in the setting of thoracic irradiation with modulated RT, the V_5 Gy_ parameter must be evaluated in addition to the DVCs established at the time of their publication [86]. Therefore, great caution is required when using “new” RT techniques with “old” DVCs. Furthermore, our bibliographic research only included papers, thus, excluding other potentially useful sources, as in the case of the textbook published by Rancati and Fiorino in 2019 [87], which offers an in-depth update of the QUANTEC initiative. In addition, other potentially useful bibliographic sources, such as those relating to the HyTEC initiative (Hygh dose per fraction, hypofractionated treatment effects in the clinic) [88,89], were deliberately excluded. In fact, our choice was to provide the most practical indications and, therefore, to include only recommendations based on dose and volume limit values or percentages.

Furthermore, both the recommendations from guidelines and from other sources may be based on previous publications and it is difficult, and sometimes impossible, to verify that these references are completely correct and, above all, provide precise indications on the clinical reference setting.

Similarly, Ferini et al. effectively summarized a large series of ever-changing dosimetric issues about the rectum, including contradictory perspectives on how to consider such an OAR (parallel vs. serial) and, consequently, conflicting DVCs [90]. In this scenario, there is also the need to understand whether some medical interventions can improve the rectal tolerance to high-radiation exposure [91,92].

Furthermore, the introduction of new algorithms to calculate dose distribution can also lead to unexpected OaR overdosing [93]. On the other hand, new techniques can improve the tolerance of some tissues. For example, Table 1 reports 72 Gy as the D_max_ value for the brain. Instead, some studies based on intensity-modulated RT or volumetric-modulated arc therapy showed the brain’s tolerability of doses up to 80 Gy [94,95]. Furthermore, many indications have rather low levels of evidence and have to be simply considered as “expert opinions". For example, all suggestions presented in Supplementary Table S3 for breast cancers have the minimum level of evidence (D). More generally, the categorization of the source of recommendation we propose has further limitations. In fact, we arbitrarily considered recommendations from guidelines as those with the highest level of evidence, considering this, even if a compromise, the best possible choice. However, as is well known, the same guidelines can be based on levels of evidence that we classify as of lower level and it is not certain that the recommendations of the guidelines are updated based on the latest data from the literature. 

Finally, the “practical” choice of producing “synoptic” guidelines further limited the presented information. For example, only the DVCs for RT delivered in one, three, five, and eight fractions were arbitrarily selected for ultra-hypofractionated treatments.

Additionally, no DVC indication is made for RT schedules combining different radiation dose sizes, as in the cases of a stereotactic boost to a limited tumor site after a more extended normofractionated irradiation [96,97] or of a large spatially fractionated dose administration before a homogeneously delivered hypofractionated palliative course [98].

## 5. Conclusions

In conclusion, we summarized, in a single document, information on the definition of the main OaRs, on the imaging modalities suggested for their optimal identification and on the selected DVCs. The authors hope to have provided a concise but useful guideline for clinical practice in RT.

## Figures and Tables

**Table 1:** General dose–volume constraints for adult patients (organ nomenclature based on the Global Quality Assurance of Radiation Therapy Clinical Trials Harmonization Group (GHG) contouring guidelines [29]).

Organ	Constraints (Conventional Fractionation) *	Constraints (Hypofractionation)			
		1 Fraction	3 Fractions	5 Fractions	8 Fractions
Anal Canal	D_MEAN_ < 40 Gy [30] (C) [31] (B) V_20 Gy_ < 75% [32] (C)				
Anterior Descending Artery	V_15 Gy_ ≤ 10% [33] (C)				
Bladder	For bladder cancer treatment: D_MAX_ < 65 Gy; For prostate cancer treatment: V_80__Gy_ < 15%; V_75__Gy_ < 25%; V_70__Gy_ < 35%; V_65__Gy_ < 50%; [5] (A); For anal cancer treatment: V_50__Gy_ < 5%; V_40__Gy_ < 35%; V_35__Gy_ < 50% [28] (A)	Dose to bladder-wall: D_MAX(0.1_ _cm^3^__)_ < 18.4 Gy [6] (A); V_11.4__Gy_ < 15 cm^3^ [8] (A)	Dose to bladder-wall: D_MAX(0.1_ _cm^3^__)_ < 28.2 Gy [6] (A); D_15__cm^3^_ < 16.8 Gy; [7] (A)	Dose to bladder-wall: D_MAX(0.1_ _cm^3^__)_ < 38 Gy [6] (A); D_15__cm^3^_ < 18.3 Gy; [7] (A) For primary prostate SBRT only: V_18.1__Gy_ < 40%; V_37__Gy_ < 10 cm^3^_(mandatory)_; V_37 Gy_ < 5 cm^3^_(optimal)_ [7] (A)	
Bone Mandible	V_50__Gy_ < 31–32% or V_50__Gy_ < 31 cm^3^ [34] (B); D_MAX_ < 70–73.5 Gy_(mandatory)_; V_55 Gy_ < 20%_(optimal)_ [35] (A)				
Bowel	V_15__Gy_ < 120 cm^3^; V_45__Gy_ < 195 cm^3^ [5] (A)			For primary prostate SBRT only: V_18.1__Gy_ < 5 cm^3^; V_30__Gy_ < 1 cm^3^_(mandatory)_ [7] (A)	
Bowel Large	V_45__Gy_ < 5% or <20 cm^3^; V_35__Gy_ < 35% or 150 cm^3^; V_30__Gy_ < 50% or 200 cm^3^ [28] (A)	D_MAX(__0.1__cm^3^__)_ < 18.4 Gy_(mandatory)_ [6] (A); V_14.3__Gy_ < 20 cm^3^_(optimal)_ [8] (A)	D_MAX(0.1_ _cm^3^__)_ < 28.2 Gy [6] (A); D_20__cm^3^_ <24 Gy_(optimal)_; [8] (A)	D_MAX(0.1_ _cm^3^__)_ < 38 Gy [6] (A); D_20__cm^3^_ < 25 Gy_(optimal)_; [8] (A)	
Bowel Small	D_MAX_ ≤ 55 Gy; V_50 Gy_ ≤ 10 cm^3^_(optimal__)_; V_15 Gy_ ≤ 120 cm^3^_(optimal__)_; V_50 Gy_ ≤ 10%; V_45 Gy_ ≤ 15% [36] (A);	D_MAX(__0.1 cm^3^__)_ < 15.4 Gy_(mandatory)_ V_11.9__Gy_ < 5 cm^3^ _(mandatory)_ [6] (A);	D_MAX(0.5_ _cm^3^__)_ < 25.2 Gy_(mandatory);_ D _5__cm^3^_ < 17.7 Gy_(mandatory)_ [6] (A);	D_MAX(0.5_ _cm^3^__)_ < 35 Gy_(mandatory)_; D_MAX(0.5_ _cm^3^__)_ < 30 Gy_(optimal)_; D_10__cm^3^_ < 25 Gy_(optimal)_; [6] (A)	
Bowel Space	V_45 Gy_ < 195 cm^3^ [5] (A)				
Brachial Plexus	D_MAX(0.1 cm^3^__)_ < 60 Gy_(optimal__)_; D_MAX(0.1 cm^3^__)_ < 66 Gy_(mandatory)_ [35] (A)	D_MAX__(__0.1 cm^3^__)_ < 15 Gy_(mandatory)_ [6] (A); D_MAX__(__0.1 cm^3^__)_ < 17.5 Gy_(mandatory)_ [25] (A); V_14 Gy_ < 3 cm^3^_(optimal__)_; [8] (A)	D_MAX__(0.1__cm^3^__)_ <24 Gy_(mandatory)_ [6,25] (A) V_20.4__Gy_ < 3 cm^3^ [8] (A)	D_MAX__(0.1__cm^3^__)_ < 30.5 Gy_(optimal)_; D_MAX__(0.1__cm^3^__)_ < 32 Gy_(mandatory)_ [6] (A)	D_MAX(__0.1__cm^3^__)_ < 35 Gy_(optimal)_; [25] (A) D_MAX__(0.1__cm^3^__)_ < 39 Gy_(mandatory)_ [6] (A)
Brain	V_60 Gy_ ≤ 3 cm^3^ [37] (A); D_MAX_ < 72 Gy; [5] (A)	Whole brain less GTV: D_50%_ < 5 Gy D_10 cm^3^_ < 12 Gy [7] (A) Brain including target: V_12 Gy_ < 10–15 cm^3^; [6] (A)	D_20__cm^3^_ < 20 Gy_(optimal)_ [6] (A); V_14 Gy_ < 7 cm^3^ [38] (C)	D_20__cm^3^_ < 24 Gy_(optimal)_ [6] (A)	
Brainstem	Brainstem PRV: D_MAX_ < 54 Gy; D_1–10__cm^3^_ < 59 Gy (peripheral edge) [5,34,39] (A)	D_MAX(0.035_ _cm^3^__)_ < 15 Gy_(mandatory)_; D_MAX(0.035_ _cm^3^__)_ < 10 Gy_(optimal)_; [6] (A)	D_Max (0.035_ _cm^3^__)_ < 23.1 Gy_(mandatory)_; D_MAX(0.035_ _cm^3^__)_ < 18 Gy_(optimal)_; [6] (A)	D_MAX(0.035_ _cm^3^__)_ < 31 Gy_(mandatory)_; D_MAX (0.035_ _cm^3^__)_ < 23 Gy_(optimal)_; [6] (A)	
Cauda Equina		D_MAX(0.035 cm^3^__)_ < 16 Gy_(mandatory)_ [6] (A); V_14 Gy_ < 5 cm^3^_(optimal)_; [8] (A)	D_MAX (0.035 cm^3^__)_ < 24 Gy_(mandatory)_ [6] (A); D_5 cm^3^_ < 21.9 Gy_(optimal__)_; [8] (A)	D_MAX (0.035 cm^3^__)_ < 32 Gy_(mandatory)_ [6] (A); D_5 cm^3^_ < 30 Gy_(optimal)_; [8] (A)	
Chestwall		D_MAX__(__0.01 cm^3^__)_ <30 Gy_(optimal)_ [6,25] (A); V_22 Gy_ < 1 cm^3^_(optimal__)_; [8] (A)	D_MAX(0.1_ _cm^3^__)_ < 36.9 Gy _(optimal)_ [6] (A); D_MAX(0.1_ _cm^3^__)_ < 30 Gy [25] (A); D_30__cm^3^_ < 30 Gy [7] (A)	D_MAX__(0.1__cm^3^__)_ < 43 Gy_(optimal)_ [6] (A); D_30__cm^3^_ < 32 Gy [7] (A)	D_MAX__(0.5__cm^3^__)_ <39 Gy; D_30__cm^3^_ < 35 Gy [7] (A)
Cochlea	Ideally one side; D_MEAN_ < 45 Gy [5,39] (A) [40] (B)	D_MEAN_ < 9 Gy _(mandatory)_ [7] (A); D_MEAN_ < 4 Gy _(optimal)_ [6] (A); D_MAX_ < 12 Gy [10] (B)	D_MEAN_ < 17.1_(optimall)_ [6] (A); D_MAX_ < 20 Gy [10] (B)	D_MEAN_ < 25 Gy_(optimal)_ [6] (A); D_MAX_ < 27.5 Gy [10] (B)	
Common Bile Duct		D_MAX(0.1 cm^3^__)_ < 30 Gy_(mandatory)_ [6] (A);	D_MAX(0.1 cm^3^__)_ < 50 Gy_(optimal)_ [6] (A);	D_MAX(0.1 cm^3^__)_ < 50 Gy_(optimal)_ [6] (A);	
Cricopharyngeal Inlet	D_MAX_ < 62 Gy [41] (C)				
Cricopharyngeal muscle	D_MAX_ < 62 Gy [41] (C)				
Duodenum	D_MAX_ ≤ 55 Gy; V_50 Gy_ ≤ 10 cm^3^_(optimal)_; V_50 Gy_ ≤ 10%; V_45 Gy_ ≤ 15% [36] (A)	D_MAX(0.1_ _cm^3^__)_ < 12.4 Gy_(mandatory)_; D_10 cm^3^_ < 9 Gy _(mandatory)_ [6] (A); D_5 cm^3^_ < 11.2 Gy _(optimal)_ [8] (A)	D_MAX(0.1_ _cm^3^__)_ < 22.2 Gy_(mandatory)_; D_10__cm^3^_ < 11.4 Gy_(mandatory)_ [6] (A); D_5__cm^3^_ < 16.5 Gy_(mandatory)_; [7] (A)	D_MAX(0.1_ _cm^3^__)_ < 35 Gy_(mandatory)_; D_MAX(0.1_ _cm^3^__)_ < 33 Gy_(optimal)_; D_10__cm^3^_ < 25 Gy _(optimal)_ [6] (A); D_1__cm^3^_ < 33 Gy; D_5__cm^3^_ < 25 Gy; D_9__cm^3^_ < 15 Gy _(optimal)_; [7] (A)	
Esophageal inlet	D_MAX_ < 45 Gy_(optimal)_; D_MAX_ < 55 Gy_(mandatory)_ [35] (A)				
Esophagus	D_MEAN_ < 34 Gy; V_35 Gy_ < 50%; V_50 Gy_ < 40%; V_70 Gy_ < 20% [5] (A);	D_MAX__(0.1__cm^3^__)_ < 15.4 Gy_(mandatory)_ [6,25] (A); D_5__cm^3^_ < 11.9 Gy _(optimal)_; [8] (A)	D_MAX__(0.1__cm^3^__)_ < 25.2 Gy_(mandatory)_ [6] (A); D_MAX__(0.1__cm^3^__)_ < 27 Gy [25] (A); D_0.5__cm^3^_ < 17.7 Gy_(optimal)_; [7,8] (A) V_21 Gy_ < 5 cm^3^ [10] (B)	D_MAX__(0.1__cm^3^__)_ <35 Gy_(mandatory)_ [6] (A); D_MAX__(0.5__cm^3^__)_ <32 Gy_(optimal)_; [7,8] (A) V_27.5 Gy_ < 5 cm^3^; V_19.5 Gy_ < 10 cm^3^ [10] (B); Avoid 105% of PTV prescription [25] (A)	D_MAX(0.1 cm^3^__)_ < 40 Gy_(mandatory)_ [6] (A)
Esophagus Superior	D_MAX_ < 55 Gy_(mandatory)_; D_MAX_ < 45 Gy_(optimal)_; [35] (A)	D_MAX(0.01_ _cm^3^__)_ < 15.4 Gy_(mandatory)_ [6] (A); D_5__cm^3^_ < 11.9 Gy _(optimal)_; [8] (A)	D_MAX(0.1_ _cm^3^__)_ < 25.2 Gy_(mandatory)_ [6] (A); D_0.5__cm^3^_ < 17.7 Gy_(optimal)_; [7,8] (A)	D_MAX(0.5_ _cm^3^__)_ <35 Gy_(mandatory)_ [6] (A); D_MAX(0.5_ _cm^3^__)_ <32 Gy_(optimal)_; [7,8] (A)	D_MAX(0.5 cm^3^__)_ < 40 Gy_(mandatory)_ [6] (A);
Eye Anterior	Lens D_MAX_ < 4 Gy [35] (A) Cornea D_MAX_ < 40 Gy [42] (B)	D_MAX(0.035_ _cm^3^__)_ < 1.5 Gy [6,7] (A)	D_MAX_ < 7 Gy_(mandatory);_ D_MAX_ < 3 Gy_(optimal)_; [10] (B)	D_MAX_ < 7 Gy _(mandatory);_ D_MAX_ < 3 Gy_(optimal)_; [10] (B)	
Eye	Macula D_MAX_ < 45 Gy [39] (A); Retina D_MAX_ < 45–50 Gy [43] (A) [40] (B)	D_MAX(0.1_ _cm^3^__)_ < 8 Gy [6,7] (A)			
Eye Posterior	Retina D_MAX_ < 45 Gy [43] (A)	D_MAX_ < 5 Gy [10] (B)	D_MAX_ < 15 Gy_(mandatory);_ D_MAX_ < 5 Gy_(optimal)_; [10] (B)	D_MAX_ < 15 Gy_(mandatory);_ D_MAX_ < 5 Gy_(optimal)_; [10] (B)	
Femoral Head-Neck	V_44 Gy_ < 5%; V_40 Gy_ < 35%; V_30 Gy_ < 50% [28] (A)	D_10__cm^3^_ < 14 Gy_(optimal)_ [6,8] (A)	D_10 cm^3^_ < 21.9 Gy_(optimal__)_ [6,7] (A)	D_10 cm3_ < 30 Gy_(optimal)_ [6,7] (A) For primary prostate SBRT only: V_14.5 Gy_ < 5% _(mandatory)_ [7] (A)	
Genitals	V_40 Gy_ < 5%; V_30 Gy_ < 35%; V_20 Gy_ < 50% [28] (A)				
Glottis	D_MEAN_ < 50 Gy_(mandatory)_; V_50 Gy_ < 27%_(mandatory)_; D_MEAN_ < 44 Gy _(optimal)_ [5] (A) [34] (B); D_MAX_ < 73.5 Gy [35] (A)				
Great Vessels		D_MAX__(0.1__cm^3^__)_ < 30 Gy_(mandatory)_ [6] (A); D_10__cm^3^_ < 31 Gy_(optimal)_; [8] (A) D_MAX_ < 37 Gy [25] (A);	D_MAX(0.1 cm^3^__)_ < 45 Gy_(mandatory)_ [6,7] (A); D_10__cm^3^_ < 39 Gy_(optimal)_; [8] (A);	D_MAX (0.1 cm^3^__)_ < 53 Gy_(mandatory)_ [6,7] (A); D_10__cm^3^_ < 47 Gy_(optimal)_; [8] (A) Avoid 105% of PTV prescription [25] (A)	D_MAX__(0.1__cm^3^__)_ < 65 Gy_(mandatory)_; D_MAX__(0.1__cm^3^__)_ < 60 Gy_(optimal)_ [6] (A);
Heart	D_MEAN_ < 26–30 Gy V_25 Gy_ < 10%; V_30 Gy_ ≤ 30%; [5,26] (A)	D_MAX__(0.03__cm^3^__)_ < 22 Gy_(mandatory)_ [8,25]; D_15__cm^3^_ < 16 Gy_(optimal)_; [8] (A)	D_MAX__(0.5__cm^3^__)_ < 26 Gy_(mandatory)_; D_MAX__(0.5__cm^3^__)_ < 24 Gy_(optimal)_; [7] (A) D_MAX_ < 30 Gy_(mandatory)_ [25] (A); D_15__cm^3^_ < 24 Gy [8] (A); V_21 Gy_ < 5 cm^3^ [10] (B)	D_MAX(0.5_ _cm^3^__)_ < 29 Gy_(mandatory)_; D_MAX(0.5_ _cm^3^__)_ < 27 Gy_(optimal)_; [7] (A) D_15__cm^3^_ < 32 Gy [8] (A) Avoid 105% of PTV prescription [25] (A)	D_MAX(0.5_ _cm^3^__)_ <60 Gy_(mandatory)_; D_MAX(0.5_ _cm^3^__)_ < 50 Gy_(optimal)_; [7] (A)
Heart and Pulmonary Artery	D_MEAN_ < 26–30 Gy V_25 Gy_ < 10%; V_30 Gy_ ≤ 30%; [5,26] (A)	D_MAX(0.1_ _cm^3^__)_ < 22 Gy_(mandatory)_ [6] (A); D_15__cm^3^_ < 16 Gy_(optimal)_; [8] (A)	D_MAX(0.1_ _cm^3^__)_ < 30 Gy_(mandatory)_; D_MAX(0.1_ _cm^3^__)_ < 26 Gy_(optimal)_ [6] (A); D_15__cm^3^_ < 24 Gy [8] (A)	D_MAX(0.1_ _cm^3^__)_ < 38 Gy_(mandatory)_; D_MAX(0.1_ _cm^3^__)_ < 29 Gy_(optimal)_ [6] (A); D15 cm^3^ < 32 Gy [8] (A)	D_MAX(0.1_ _cm^3^__)_ < 46 Gy_(mandatory)_; D_MAX(0.1_ _cm^3^__)_ < 40 Gy_(optimal)_ [6] (A);
Hippocampus	If HA-WBRT: D_MAX_ 16 Gy; D_100%_ < 9 Gy [44,45] (D) If HA-WBRT or PCI: D_MAX_ < 12 Gy; V_3 Gy_ ≤ 20%; [40] (B); For primary CNS tumors: V_7.2 Gy_ ≤ 40%; D_MEAN_ < 30 Gy [40] (B); [44,46] (C)				
Jejunum-Ileum	D_MAX_ ≤ 55 Gy; V_50 Gy_ ≤ 10 cm^3^_(optimal__)_; V_15 Gy_ ≤ 120 cm^3^_(optimal__)_; V_50 Gy_ ≤ 10%; V_45 Gy_ ≤ 15% [36] (A);	D_MAX(__0.1 cm^3^__)_ <15.4 Gy_(mandatory)_ V_11.9 Gy_ < 5 cm^3^ _(mandatory)_ [6] (A)	D_MAX(0.5_ _cm^3^__)_ < 25.2 Gy_(mandatory)_ D_5__cm^3^_ < 17.7 Gy_(mandatory)_ [6] (A)	D_MAX(0.5_ _cm^3^__)_ < 35 Gy_(mandatory)_; D_MAX(0.5_ _cm^3^__)_ < 30 Gy_(optimal)_; D_10__cm^3^_ < 25 Gy_(optimal)_ [6] (A)	
Kidneys	D_MEAN_ < 18 Gy [26,36] (A) V_20 Gy_ ≤ 33% [26] (A); If solitary kidney: V_18 Gy_ < 15%; V_14 Gy_ < 30% [47] (A)	V_10 Gy_ < 33% _(mandatoryl)_ [6] (A) V_8.4 Gy_ < 200 cm^3^ [10] (B)	D_200 cm^3^_ < 16 Gy_(mandatory)_ [7] (A); V_10 Gy_ < 33% _(mandatory)_ [6] (A)	If solitary kidney: V_10 Gy_ < 45%_(mandatory)_; V_10 Gy_ < 10% _(optimal)_ [6] (A) D_MEAN_ < 10 Gy_(optimal)_ [7] (A) V_17.5 Gy_ < 200 cm^3^ [10] (B)	
Kidney Cortex		D_200 cm^3^_ < 8.4 Gy [6,8] (A) If solitary kidney: V_10 Gy_ < 33%_(mandatory)_; [6] (A)	D_MEAN_ < 8.5 Gy_(optimal)_ [6] (A) D_200 cm^3^_ < 16 Gy [6,8] (A) If solitary kidney: V_10 Gy_ < 33%_(mandatory)_; [6] (A)	D_MEAN_ < 10 Gy_(optimal)_ [6] (A) D_200 cm^3^_ < 17.5 Gy [6,8] (A) If solitary kidney: V_10 Gy_ < 45%_(mandatory)_; V_10 Gy_ < 10%_(optimal)_ [6] (A)	
Lacrimal Gland	D_MAX_ < 40 Gy [40] (B); D_MEAN_ ≤ 26 Gy [35] (A)	D_MAX_ < 5 Gy [10] (B)	D_MAX_ < 15 Gy_(mandatory);_ D_MAX_ <5 Gy_(optimal)_; [10] (B)	D_MAX_ < 15 Gy_(mandatory);_ D_MAX_ < 5 Gy_(optimal)_; [10] (B)	
Larynx	D_MAX_ < 50 Gy [35] (A); D_MAX_ < 66 Gy; D_MEAN_ < 50 Gy; V_50 Gy_ < 27% [5] (A)	V_10.5 Gy_ < 4 cm^3^; V_20.2 Gy_ < 0.035 cm^3^ [10] (B)			
Lens	D_MAX_ <4 Gy [35] (A)	D_MAX(0.035_ _cm^3^__)_ < 1.5 Gy [6] (A)			
Lips	D_MEAN_ 30 Gy_(optimal)_; D_MEAN_ 50 Gy_(mandatory)_ [42] (B)				
Liver	D_MEAN_< 25 Gy [26,36] (A); V_30 Gy_ ≤ 33% [26] (A)	D_700 cm^3^_ < 9.1 Gy; [6,8] (A) V_12 Gy_ < 30%; V_5 Gy_ < 50%; V_2.5 Gy_ < 70%; [10] (B)	D_MEAN_ < 15 Gy_(mandatory);_ D_MEAN_ < 13 Gy_(optimal_; D_700 cm^3^_ < 17 Gy_(mandatory)_; D_700 cm^3^_ < 15 Gy_(optimal__)_ [6] (A); D_50%_ < 15 Gy _(optimal)_; [7] (A)	D_MEAN_ < 15.2 Gy_(mandatory);_ D_MEAN_ < 13 Gy_(optimal_; V_10 Gy_ < 70%_(optimal)_ [6,7] (A); D_700 cm^3^_ < 15 Gy; [6] (A)	
Lumbo-sacral Plexus	Pudendal Nerve D_MAX_ < 60 Gy [48] (C)	D_MAX(__0.1__cm^3^__)_ < 16 Gy_(mandatory)_; D_5 cm^3^_ < 14.4 Gy_(optimal__)_; [6,7] (A)	D_MAX(0.1 cm^3^__)_ < 24 Gy _mandatory)_; D_5 cm^3^_ < 22 Gy_(optimal__)_; [6,7] (A)	D_MAX(0.1 cm^3^__)_ < 32 Gy_(mandatory)_; D_5 cm^3^_ < 30 Gy_(optimal__)_; [6,8] (A)	
Lung	V_40 Gy_ ≤ 10%; V_30 Gy_ ≤ 15%; V_20 Gy_ ≤ 20%; V_10 Gy_ ≤ 40%; V_5 Gy_ ≤ 50%; D_MEAN_ < 20 Gy [26] (A)	Lungs and Lungs–ITV: V_20 Gy_ < 15% _(mandatory)_; D_MEAN_ < 8 Gy _(optimal)_; V_20 Gy_ < 10% _(optimal)_; [6] (A) D_1500 cm^3^_ < 7 Gy; D_1000 cm^3^_ < 7.4 Gy; [8] (A)	Lungs and Lungs–ITV: V_20 Gy_ < 15% _(mandatory)_; D_MEAN_ < 8 Gy _(optimal)_; V_20 Gy_ < 10% _(optimal)_; [6] (A) D_1500 cm^3^_ < 11.6 Gy; D_1000 cm^3^_ < 12.4 Gy; [8] (A)	Lungs and Lungs–ITV: V_20 Gy_ < 15% _(mandatory)_; D_MEAN_ < 8 Gy _(optimal)_; V_20 Gy_ < 10% _(optimal)_; [6] (A) D_1500 cm^3^_ < 12.5 Gy; D_1000 cm^3^_ < 13.5 Gy; [8] (A)	Lungs and Lungs–ITV: V_20 Gy_ < 15% _(mandatory)_; D_MEAN_ < 8 Gy _(optimal)_; V_20 Gy_ < 10% _(optimal)_; [6] (A)
Optic Chiasm	D_MAX_ < 55 Gy [39] (A)				
Optic Nerve	D_MAX_ < 55 Gy [39] (A)	D_MAX(0.035 cm^3^__)_ < 10 Gy_(mandatory)_; D_MAX(0.035 cm^3^__)_ < 8 Gy_(optimal)_; [6] (A)	D_MAX(0.035 cm^3^__)_ < 20 Gy _(mandatory)_; D_MAX(0.035 cm^3^__)_ < 15 Gy_(optimal)_; [6] (A) V_10.5 Gy_ < 0.5 cm^3^ [10] (B)	D_MAX(0.035 cm^3^__)_ < 25 Gy _(mandatory)_; D_MAX(0.035 cm^3^__)_ < 22.5 Gy _(optimal)_; [6] (A) V_12.5 Gy_ < 0.5 cm^3^ [10] (B)	
Oral Cavity	V_30 Gy_ < 73% and limit D_MEAN_ to uninvolved oral cavity [34] (B)				
Ovaries	D_MEAN_ < 8.8 Gy [49] (D); D_MEAN_ < 15 Gy [50] (D); V_7.5 Gy_ < 26% [51] (C)	;			
Pancreas	Limit V_45 Gy_ [52] (C); D_MEAN_ < 25 Gy [53] (D)				
Parotid Gland	D_MEAN_ < 25 Gy for both glands; D_MEAN_ < 20 Gy for single gland [5,42] (A); V_30 Gy_ < 50%; V_40 Gy_ < 33% _(contralateral)_ [35] (A)				
Penile Bulb	V_90 Gy_ < 50%; V_60–70 Gy_ < 70% [5] (A)	D_MAX(__0.03__cm^3^__)_ < 34 Gy_(mandatory)_; D_MAX(3 cm^3^__)_ < 14 Gy_(optimal__)_; [8] (A)	D_MAX(3 cm^3^__)_ < 42 Gy_(mandatory)_; D_MAX(3 cm^3^__)_ < 21.9 Gy_(optimal__)_; [7,8] (A)	D_MAX(3 cm^3^__)_ < 50 Gy_(mandatory)_; D_MAX(3 cm^3^__)_ < 30 Gy_(optimal__)_; [7,8] (A) For primary prostate SBRT only: V_29.5 Gy_ < 50% _(mandatory)_ [7] (A)	
Pharyngeal constrictor muscles	D_MEAN_ < 50 Gy [35,42] (A)				
Pharyngeal constrictor muscles Inferior	D_MEAN_ < 50 Gy [35,42] (A)				
Pharyngeal constrictor muscle Middle	D_MEAN_ < 50 Gy [35,42] (A) V_50–60 Gy_ < 70% [34] (B)				
Pharyngeal constrictor muscle Superior	D_MEAN_ < 50 Gy [35,42] (A) V_50–60 Gy_ < 70% [34] (B)				
Pituitary Fossa	Pituitary: D_MAX_ < 50 Gy [39] (A) [40] (B)	D_MEAN_ < 9 Gy [54] (B) †			
Pituitary Gland	D_MAX_ < 50 Gy [39] (A) [40] (B)				
Proximal Bronchus		D_MAX__(0.1 cm^3^__)_ < 20.2 Gy _mandatory)_ [6,25] (A); D_4 cm^3^_ < 10.5 Gy; [8] (A)	D_MAX__(0.1 cm^3^__)_ < 30 Gy _mandatory)_ [6] (A); D_MAX__(0.5 cm^3^__)_ <30 Gy_(optimal)_; [7,25] (A); D_4 cm^3^_ < 15; [8] (A)	D_MAX__(0.1 cm^3^__)_ < 38 Gy _mandatory)_; D_MAX__(0.1 cm^3^__)_ < 35 Gy_(optimal)_; [6] (A); D_4 cm^3^_ < 16.5; [8] (A); Avoid 105% of PTV prescription [25] (A)	D_MAX__(0.1 cm^3^__)_ < 40 Gy_(mandatory)_; [6] (A); D_MAX__(0.5 cm^3^__)_ < 32 Gy_(optimal)_; [7] (A)
Rectum	V_50 Gy_ < 50%; V_60 Gy_ < 35%; V_65 Gy_ < 25%; V_70 Gy_ < 20%; V_75 Gy_ < 15% [5] (A)	D_MAX(0.01 cm^3^__)_ < 18.4 Gy_(mandatory)_ [6] (A); D_20 cm^3^_ < 14.3 Gy [8] (A)	D_MAX(0.1 cm cm^3^__)_ < 28.2 Gy [6] (A); D_20 cm^3^_ < 24 Gy [8] (A)	D_MAX(0.1 cm^3^__)_ < 38 Gy [6] (A); D_20 cm^3^_ < 25 Gy [8] (A) For primary prostate SBRT only: V_18.1 Gy_ < 50% V_29 Gy_ < 20% V_36 Gy_ < 1 cm^3^_(mandatory)_ [7] (A)	
Retina	D_MAX_ < 45–50 Gy [37] (A)				
Sigmoid Colon		D_20 cm^3^_ < 18.4 Gy _(mandatory)_; D_20 cm^3^_ < 14.3 Gy _(optimal)_; [8] (A)	D_20 cm^3^_ < 28.2 Gy_(mandatory)_; D_20 cm^3^_ < 24 Gy_(optimal__)_; [8] (A)	D_20 cm^3^_ < 38 Gy_(mandatory)_; D_20 cm^3^_ < 25 Gy_(optimal__)_; [8] (A)	
Skin	D_0.03 cm^3^_ ≤ 25 Gy [43] (A) ‡	D_MAX__(0.01 cm^3^__)_ < 26 Gy_(mandatory)_ [6,25] (A); D_10 cm^3^_ < 23 Gy_(mandatory)_ [6] (A)	D_MAX__(0.1 cm^3^__)_ <33 Gy_(optimal)_; D_10 cm^3^_ < 30 Gy_(optimal)_ [6] (A); D_MAX_ < 24 Gy [25] (A)	D_MAX__(0.1 cm^3^__)_ < 39.5 Gy_(mandatory)_; D_10 cm^3^_ < 36.5 Gy_(mandatory)_ [6] (A); D_MAX_ < 32 Gy [25] (A)	D_MAX__(0.1 cm^3^__)_ < 48 Gy_(optimal)_; D_10 cm^3^_ < 44 Gy_(optimal)_ [6] (A)
Spinal Canal	D_MAX_ < 45–50 Gy [5,26] (A)	D_MAX__(0.035 cm^3^__)_ < 14 Gy_(mandatory)_ [6,25] (A) D_MAX__(0.035 cm^3^__)_ < 12.4 Gy_(optimal)_; [6] (A)	D_MAX__(0.035 cm^3^__)_ < 20.3 Gy_(mandatory)_ [6] (A); D_MAX__(0.1 cm^3^__)_ < 18 Gy_(optimal)_; [7,8,25] (A)	D_MAX__(0.035 cm^3^__)_ < 25.3 Gy_(mandatory)_ [6] (A); D_MAX_ < 30_(mandatory)_ Gy [7,25] (A)	D_MAX__(0.035 cm^3^__)_ < 32 Gy_(mandatory)_ [6] (A) D_MAX__(0.1 cm^3^__)_ < 25 Gy_(optimal)_; [7] (A)
Spinal Cord	D_MAX_ < 45–50 Gy [5,26] (A)	D_MAX__(0.035 cm^3^__)_ < 14 Gy_(mandatory)_ [6,25] (A) D_MAX__(0.035 cm^3^__)_ < 12.4 Gy_(optimal)_; [6] (A)	D_MAX__(0.035 cm^3^__)_ < 20.3 Gy_(mandatory)_ [6] (A); D_MAX__(0.1 cm^3^__)_ < 18 Gy_(optimal)_; [7,8,25] (A)	D_MAX__(0.035 cm^3^__)_ < 25.3 Gy_(mandatory)_ [6] (A); D_MAX_ < 30_(mandatory)_ Gy [7,25] (A)	D_MAX__(0.035 cm^3^__)_ < 32 Gy_(mandatory)_ [6] (A) D_MAX__(0.1 cm^3^__)_ < 25 Gy_(optimal)_; [7] (A)
Spleen	D_MEAN_ < 8.8 Gy; V_5 Gy_ < 30.0%; V_10 Gy_ < 30.0% V_15 Gy_ < 20.0% V_20 Gy_ < 20.0% [55,56,57] (C)				
Stomach	D_MAX_ < 54 Gy; D_MEAN_ < 45 Gy [26] (A); V_45 Gy_ ≤ 75 cm3_(optimal)_; V_50 Gy_ ≤ 10%; V_45 Gy_ ≤ 15% [36] (A);	D_MAX__(0.1 cm^3^__)_ < 12.4 Gy_(mandatory)_ [6,25] (A); D_10 cm^3^_ < 11.2 Gy _(optimal)_ [6,8] (A)	D_MAX__(0.1 cm^3^__)_ < 22.2 Gy_(mandatory)_; D_10 cm^3^_ < 16.5 Gy_(mandatory)_ [6] (A)	D_MAX__(0.1 cm^3^__)_ < 35 Gy_(mandatory)_; D_MAX__(0.1 cm^3^__)_ < 33 Gy_(optimal)_; D_10 cm^3^_ < 25 Gy_(optimal)_; D_50 cm^3^_ < 12 Gy _(optimal)_ [6] (A)	
Submandibular Gland	D_MEAN_ < 35 Gy [35] (A)				
Supraglottic larynx	D_MAX_ < 66 Gy [35] (A)				
Temporal Lobe	D_MAX_ < 65 Gy; D_MAX(1 cm^3^__)_ < 60 Gy_(mandatory)_ [35] (A)				
Testis	In case of TBI: D_MAX_ < 6 Gy D_MEAN_ < 5 Gy [58,59] (D)			For primary prostate SBRT only: Avoid beam entry [7] (A)	
Thyroid Gland	V_45 Gy_ < 50% [35] (A); D_MEAN_ < 45 Gy or sparing at least 5 cm^3^ of the thyroid < 45 Gy [34] (B)				
Trachea		D_MAX(__0.1 cm^3^__)_ < 20.2 Gy_(mandatory)_ [6,25] (A); D_4 cm^3^_ < 10.5 Gy [8] (A)	D_MAX__(0.1 cm^3^__)_ < 30 Gy_(mandatory)_ [6,25] (A); D_4 cm^3^_ < 15 Gy [8] (A)	D_MAX__(0.1 cm^3^__)_ < 38 Gy _mandatory)_; D_MAX__(0.1 cm^3^__)_ < 35 Gy_(optimal)_ [6] (A); D_4 cm^3^_ < 16.5 Gy; [8] (A) Avoid 105% of PTV prescription [25] (A)	D_MAX__(0.1 cm^3^__)_ < 40 Gy_(mandatory)_ [6] (A); D_MAX__(0.5 cm^3^__)_ < 32 Gy_(optimal)_; [7] (A)
Ureters		D_MAX__(0.1 cm^3^__)_ < 35 Gy [6] (A)	D_MAX__(0.1 cm^3^__)_ <40 Gy [6] (A)	D_MAX_ _(0.5 cm^3^__)_ <45 Gy [7] (A)	
Urethra	In case of EBRT + BRT: D_0.1 cm^3^_ ≤ 120 Gy _EQD2_ [60] (A) D_10 cm^3^_ ≤ 120 Gy _EQD2_; D_30 cm^3^_ ≤ 105 Gy _EQD2_; [60,61] (A)			V_47 Gy_ < 20% [10] (B)	
Prostatic Urethra	In case of EBRT + BRT: D_0.1 cm^3^_ ≤ 120 Gy _EQD2_ [60] (A) D_10 cm^3^_ ≤ 120 Gy _EQD2_; D_30 cm^3^_ ≤ 105 Gy _EQD2_; [60,61] (A)			For primary prostate SBRT only: D_50%_ < 42 Gy _(optimal)_ [7] (A)	

**Legend:** BRT: Brachytherapy, CNS: central nervous system; DMax: Maximum Dose; DMean: Mean Dose; DVH: Dose–volume Histogram; EBRT: External Beam Radiation Therapy, EQD2: equivalent total dose in 2-Gy fractions; GTV: Gross Tumour Volume; Gy: Gray; GyE: Gray Equivalent; HA-WBRT: Hippocampal avoidance—whole-brain radiation therapy; MRI: Magnetic resonance imaging; OAR: Organ at Risk; PCI: prophylactic cranial irradiation; PRV: planning risk volume; SBRT: stereotactic body radiation therapy; TBI: Total Body Irradiation. Common abbreviations were used in the tables: V = Volume receiving a dose ≥ Gy, D = dose received by % of the organ volume, D = dose received by γ cm^3^ (the cubic centimeters) of the organ volume, DMAX = maximum dose received by the organ, DMEAN = mean dose received by the organ. Volumes and doses were ex-pressed as percentage (%) or absolute values (cm^3^ or Gy, respectively). The letters in brackets indicate the source of recommendation, classified as follows: (A) international guidelines; (B) literature review on clinical or planning studies; (C) data from results of clinical or planning studies; (D) expert opinions or used in prospective trials. * considered 180–200 cGy/fraction, as defined by Marks et al. (3); † From a literature review. Pooled Incidence of New hormone deficiency after gamma knife radio-surgery was 8% at 5-years. ‡ constraint to avoid local permanent alopecia.

**Table 2:** Anatomical description of organs at risk (organ nomenclature based on the Global Quality Assurance of Radiation Therapy Clinical Trials Harmonization Group (GHG) contouring guidelines [29]).

Organ	Imaging Technique	Anatomical Description
Anal Canal {Canal_Anal}		Consider from the anorectal junction to the anal verge [62] [γ]. Include the internal and external anal sphincters. - Internal anal sphincter: thin muscle, which encircles the anal canal from anal mucosa (inner) to external anal sphincter (outer). - External anal sphincter: surrounds the internal anal sphincter, cranially merged with elevator ani muscles; limited by the central perineal tendon [30] [δ].
Anterior Descending Artery {A_LAD}		Descending in the anterior inter-ventricular groove to the apex of the heart. Proximal: The proximal 1/5th of the vessel, from the end of the left main coronary artery passing anteriorly behind the pulmonary artery. Mid: The mid 2/5th of the vessel descending anterolaterally in the anterior interventricular groove. Distal: The distal 2/5th of the vessel running in the interventricular groove and extending to the apex [29,63,64] [α].
Aorta {Aorta}		Divide into: thoracic aorta (ascending aorta, aortic arch, descending aorta) and abdominal aorta. Thoracic aorta: from aortic valve (III intercostal space), the diaphragmatic crurae (anterior to the T12 vertebral body). Abdominal aorta: from diaphragmatic crura to common iliac arteries bifurcation (L4 vertebral body) [62] [γ].
Aortic valve		The aortic valve is located between the left ventricle and aorta. It is a semilunar valve, posterior to the pulmonary valve. It is composed of three cups: the left posterior (origin of left coronary), anterior (origin of the right coronary), and right posterior [62] [γ].
Bichat’s Fat pad		It is located on either side of the face, between the buccinator muscle and the masseter, the zygomaticus major, and the zygomaticus minor muscles. Composed in three lobes, anterior, intermediate, and posterior. It also has four extensions: sublevator, melolabial, buccal, and pterygoid, whose names derived from their location and proximal muscles [65] [γ].
Bladder {Bladder}	MRI or CT	Inferiorly from its base and superiorly to the dome. Include all the layers and any content [66] [α].
Bone Mandible {Bone_Mandible}	CT-bone window	Consider the entire mandible bone, from the temporo-mandibular joint to the symphysis mandibular. Exclude the teeth from the contour [67] [α].
Bone Marrow {Bone_Marrow}		Pelvic Bone Marrow (BM) is divided into three subsites: - Ilium: from the iliac crests extending to the superior border of the femoral heads; - Lower pelvis: consisting of the pubes, ischia, acetabula, and proximal femora extending from the superior border of the femoral heads to the inferior border of the ischial tuberosities; - Lumbosacral spine (LSS): from the most superior vertebral body contained in the planning treatment volume (usually L5) inferiorly to include the entire sacrum [68] [δ].
Bowel {Bowel}	CT with oral contrast	Consider duodenum, jejunum and ileum (small bowel), and large bowel (caecum, colon until sigmoid colon) as single volume. Delineate the bowel loops from the pylorus to the recto-sigmoid junction, limiting closely to the external bowel wall. Include bowel contents in the contour [29] [α].
Bowel Large {Bowel_Large}		Include portions of all the ascending, transverse, descending, and sigmoid colon. Caudal limit: rectosigmoid junction. The presence of haustra, sacculations, and appendices epiploicae may help to distinguish large bowel from small bowel. Contour all the mucosal layers and include bowel contents [29,66] [α].
Bowel Small {Bowel_Small}	CT with oral contrast	It includes duodenum, jejunum, and ileum. Contour from pylorus to the ileocaecal junction. Track the bowel slice by slice without the intertwining mesentery. The presence of valvulae conniventes and bowel contents may help to distinguish from large bowel. Contour all the mucosal layers and include bowel contents. The use of oral contrast may help to distinguish bowel loops [29,66] [α].
Bowel Space {Spc_Bowel}		The bowel space represents the volume occupied by bowel loops from the level of the pylorus to the recto-sigmoid junction. Incorporate all portions of the peritoneal cavity aside from non-bowel structures. Useful if no oral contrast is used. Non-recommended in place of small and large bowel contouring if D_max_ to the large and small bowel is clinically relevant [29,66,69] [α].
Brachial Plexus {BrachialPlex_L BrachialPlex_R BrachialPlexs}		Proximal: ventral rami of cervical nerve roots at the level of C5. Distal: nerve roots al the level of T1. Then located between the anterior and middle scalene muscles to the subclavian artery; it continues then laterally into the axilla. Delineate each brachial plexus separately. *BrachialPlexs,* the sum of two volumes, may also be considered [29,67] [α].
Brain {Brain}		Include Cerebellum, Cerebrospinal fluid, and small brain vessels; exclude the brainstem and large cerebellar vessels (sigmoid sinus, transverse sinus, and superior sagittal sinus) [29,37,67] [α].
Brainstem {Brainstem}	MRI	Divided into three parts: midbrain, pons, and medulla oblongata. Midbrain: from the nigral substance at the cerebral peduncle to the upper border of the pons. Include the quadrigeminal plate. Pons: caudal to midbrain, an oval-shaped structure on sagittal views. Medulla oblongata: from the pons to the dens of C2 [29,37,39] [α].
Breast {Breast_L Breast_R Breasts}		Cranial: Upper border of palpable/visible breast tissue; maximally up to the inferior edge of the sternoclavicular joint. Caudal: Most caudal CT slice with visible breast. Ventral: 5 mm under skin surface. Dorsal: Major pectoral muscle or costae and intercostal muscles. Medial: Lateral to the medial perforating mammalian vessels; maximally to the edge of the sternal bone. Lateral: Lateral breast fold; anterior to the lateral thoracic artery Exclude skin and rib from contour. *Breasts* may be considered as sum of two volumes [29,70] [α].
Cauda Equina {CaudaEquina}	T1-weighted and T2-weighted MRI	Consider the thecal sac within the spinal canal. Cranial edge: at the level of L1–2. Caudal edge: at the level of S1. Do not contour individual intrathecal nerves within the Thecal Sac. If including the bony canal, consider caudal to the termination of the Thecal Sac, up to the inferior aspect of the S5 vertebra. No Planning organ-at-risk volume is required [29] [α] [71,72] [δ].
Chestwall {Chestwall_L Chestwall_R Chestwall}		Contour each structure separately. Include the intercostal muscles and other muscles, from lateral edge of the sternum, until the lateral edge of vertebral body. Exclude the skin from the contour. Consider a possible auto-segmentation from the corrected lung edges with a 2 cm expansion in the lateral, anterior, and posterior directions. *Chestwall* may be also considered as sum of the two volumes [29,73] [α].
Cochlea {Cochlea_L Cochlea_R Cochlea}	CT—bone windows	Consider each structure separately. Located in a bony cavity in the petrous portion of the temporal bone. It has a spiral structure and continues cranially with the semicircular canals, laterally with the internal auditory canal. *Cochlea* may also be considered as sum of the two volumes [29,37,39] [α].
Common Bile Duct {BileDuct_Common}		Cranial: first bifurcation or at the entry to the portal triad. Caudal: union with the pancreatic duct to form the ampulla of Vater [29,69] [α].
Coronary vessels		The left coronary artery is divided into Left Main Coronary artery (LMCA), Left Anterior Descending Artery (A_LAD, divided in three segments), and (CxCA, divided in two segments). The right coronary artery (RCA) is divided in 4 segments and the posterior descending artery (PDA). [74] (A) Consider the specific anatomical descriptions reported for individual coronary arteries.
Cricopharyngeal Inlet {Inlet_Cricophar}		It included the cricopharyngeal muscle and the proper esophageal inlet. For the cricopharyngeal inlet: cranial edge: arytenoid cartilage, caudal edge: consider 10mm caudal to the lower edge of the cricoid cartilage, anterior edge: cricoid cartilages (posterior limit), posterior edge: prevertebral muscle, lateral edge: thyroid cartilage, fatty tissue, and thyroid gland [29,67] [α] [75] [β].
Cricopharyngeal muscle {Musc_Cricophar}		Cranial edge: arytenoid cartilages. Caudal edge: cricoid cartilages. Anterior edge: cricoid cartilage (posterior limit). Posterior edge: pre-vertebral muscles. Lateral edge: thyroid cartilage thyroid gland [29] [α] [73] [β].
Duodenum {Duodenum}		First part: consider the pylorus and it is suspended by the hepatoduodenal ligament. Posterior limit: Common Bile Duct, Portal Vein, and Inferior Vena Cava. Second (descending) part: attached to the head of the pancreas. Third (transverse) portion: at the level of L3, it is placed between aorta and Inferior Vena Cava (anteriorly) and the Superior Mesenteric Artery and Vein (posteriorly). Fourth (ascending) part: at the level of L3, it is limited distally by the ligament of Treitz, marking the end of the duodenum. [29,69] [α]
Esophageal inlet {Inlet_Esophagus}	CT—mediastinal windows	Consider from the caudal edge of the cricoid cartilage, it extends 10mm cranio-caudally to the cervical esophagus [29,67] [α] [41] [δ].
Esophagus {Esophagus}	CT—mediastinal windows	Include all the layers of the wall. Begin at the level of cricoid cartilage until the gastroesophageal junction until it ends at the stomach [29,73] [α].
Esophagus Superior {Esophagus_S}	CT—mediastinal windows	Cranial Limit: 1 cm caudal to the lower edge of the cricoid cartilage. Caudal limit: caudal edge of C7 [67,73] [α].
Eye Anterior {Eye_A_L Eye_A_R}	MRI or CT	Consider the anterior segment of the eye, it includes: cornea, iris, ciliary body, and lens. Do not include the extra-ocular muscles in the contour [29,67] [α].
Eye {Eye_L Eye_R Eyes}	MRI or CT	Eye: consider the whole of the outside of the globe, include sclera and cornea [39] [α]. Retina: neurosensorial membrane located at the posterior part of the eyeball. It is the deepest of the three layers that form the wall of the eyeball (sclera, uvea/choroid, and retina). Anterior border of the retina: insertion of the medial rectus muscle and the lateral rectus muscle, posterior to the ciliary body. Exclude the optic nerve from the contour [37] [α].
Eye Posterior {Eye_P_L Eye_P_R}	MRI or CT	Consider the posterior segment of the eye: it includes the anterior hyaloid membrane, vitreous humor, retina, and choroid. See the anatomic description of *Retina*. Do not include the optic nerve and extra-ocular muscles [67] [α].
Femoral Head-Neck {FemurHeadNeck_L FemurHeadNeck_R}	CT—bone windows	Cranial: top of the ball of the femur; Caudal: the lowest level of the ischial tuberosity (right or left) and the cranial edge of lesser trocanters. Contour each femoral head and neck separately [29,66,76] [α].
Femur		The femur is the only bone in the upper leg. It is classified as a long bone and is normally divided into diaphysis (or body) and two epiphyses (ends). The proximal end contains the head, neck, two trochanters, and adjacent structures. The body of the femur is thick and almost cylindrical in shape. The lower end of the femur is the thickest and ends with two condyles that articulate with the tibia. [62] [γ] The diaphysis cross-sectioned by the beam entrances. Reduce the femur dose using VMAT [77] [δ].
Genitals {Genitals}	MRI	In males: include the penis, scrotum, and area including skin and fat anterior to the pubic symphysis. In females: include the clitoris, labia majora and minora, and area including skin and fat anterior to pubic symphysis. Cranial limit: caudal edge of the pubic symphysis [29,76] [α].
Glottis {Glottis}		Include the vocal cords and paraglottic fat. Cranial edge: arytenoid cartilages, caudal edge: thyroid cartilage (anterior part) Posterior edge: cricoid cartilage and arytenoid cartilages (anterior border). Exclude the air from the contour [29,67] [α] [75] [β].
Great Vessels {GreatVes}	CT—mediastinal windows	They are defined as major arteries and veins that convey blood to the heart or away from the heart. They include: aorta, pulmonary artery, pulmonary veins, superior vena cava, and inferior vena cava. The branches and tributaries of these named vessels (e.g., brachiocephalic trunk, bra-chiocephalic veins) may be included. The use of intravenous contrast may help to distinguish from adjacent mediastinal structures [29] [α] [62,78] [γ].
Heart {Heart}	CT—mediastinal windows	Contour the heart along with the pericardial sac. Cranial edge (or base): at the bifurcation of the pulmonary trunk and right pulmonary artery. Caudal edge: apex of the heart. Exclude major vessels from the contour [29,63] [α].
Heart and Pulmonary Artery {Heart + A_Pulm}	CT—mediastinal windows	See the anatomic description of *Heart*. Consider as cranial limit the cranial aspect of the pulmonary artery [29] [α].
Hearth—PTV {Heart-PTV}		Consider the anatomical description of *{Heart}.* Then, subtract the Planning Target Volume (PTV) to estimate the dose to the residual Heart. Consider this Volume for Stereotactic Arrhythmia Radioablation (STAR) [22] [δ].
Hippocampus {Hippocampus_L Hippocampus_R Hippocampi}	T1-weighted MRI	Composed by a posterior corpus and anterior head delimited by the lateral ventricle. Medial edge: quadrigeminal cistern. Lateral edge: temporal horn of the lateral ventricle. Dorsal edge: uncus. Contour each hippocampus separately. *Hippocampi* may be considered as volume sum for reporting purposes [29,37] [α] [40,79] [β].
Hypothalamus {Hypothalamus}		Composed by two separate volumes on each side of the third ventricle. Superior boundaries: anterior and the posterior commissure. Inferior boundary: base of the third ventricle. Posterior boundary: interpeduncular fossa. Medial Border: third ventricle or the visible Cerebrospinal Fluid space. Lateral border: not clearly visible, consider 3 mm from the third ventricle. Include the mammillary bodies in the contour [37] [α].
Implantable Cardioverter Defibrillator (ICD)		Do not place the ICD in the direct beam. The absorbed dose to be received by the device should be estimated before treatment [80] [α].
Jejunum-Ileum {Jejunum_Ileum}		Consider the structure from the ligament of Treitz (duodenojejunal junction) to the ileocaecal junction. Contour closely to the outer boundary. Include bowel contents [29,69] [α] [62] [γ].
Kidneys {Kidney_L Kidney_R Kidneys}		Outline kidney separately to evaluate individual dose. Consider only the organ, located from the level of the T12 and L3 vertebral bodies. Exclude the surrounding adipose tissue, any cysts, and the adrenal gland. Consider *kidney cortex* as a possible separate structure. *Kidneys* may be considered as a sum of the two volumes, which can be created to calculate total kidney dose [29,69] [α].
Kidney Cortex {Kidney-Cortex}		Consider the outer layer of renal parenchyma, under the capsule. Include also the renal columns [29] [α] [62] [γ].
Lacrimal Gland {Glnd_Lacrimal_L Glnd_Lacrimal_R}	CT—soft tissue windows	Located in the orbit superior-lateral to the eye, medially to the zygomatic process of the frontal bone. Medial border: superior rectus muscle. Inferior border: lateral rectus muscle. Contour each gland separately [29,37] [α].
Larynx {Larynx}		Composed by supraglottic and glottic components, such as: epiglottis, supraglottic adductor muscles, aryepiglottic folds, arytenoid cartilages, and the true and false vocal cords. Cranial edge: tip of the epiglottis, Caudal edge: thyroid cartilage (caudal limit). Anterior edge: hyoid bone, preepiglottic space, and thyroid cartilage. Posterior edge: The inferior pharyngeal constrictor muscles, pharyngeal lumen, and cricoid cartilage [29,67] [α].
Left Circumplex Coronary Artery (CxCA)		Proximal: from the LMCA in the left atrioventricular groove, it runs approximately 1.5 cm. Consider as caudal limit when it reaches the position between left ventricle and left atrium. Distal: from the proximal part it runs in the left atrioventricular groove in close relation to left atrium, cranially, and the inferior segment of left ventricle, caudally, to the crux of the heart [64,74] [α].
Left Main Coronary artery (LMCA)		It arises from the aorta above the aortic valve, then it passes between the pulmonary trunk and the superior part of left atrium for 10–25 mm and it divides into left anterior descended artery (LADCA) and left circumflex artery (CxCA) [64,74] [α].
Left ventricle		Cranial: consider the point after the bifurcation of LMCA. Caudal: it merges with the diaphragm. Ventral: consider an imaginary straight line from the anterior interventricular groove to the interatrial septum. Dorsal: left atrium and left atrioventricular groove cranially. Consider the pericardium caudally. Left: pericardium. Right: Cranially aorta to below the aortic ostium and caudally consider the left part of the crux [7] [α].
Lens {Lens_L Lens_R}	CT	It is a visible biconvex avascular structure, located between the vitreous humor. The position is not fixed and can vary during treatment [29,37] [α] [40] [β].
Lips {Lips}	Radiopaque marker on the surface	Cranial edge: nasal columella (cranial limit). Caudal edge: mandibular body (cranial border). Lateral edge: lateral commissure. Include the inner surface of the lip in the contour [29,67] [α] [62] [ γ].
Liver {Liver}		Consider all the 8 hepatic segments. Exclude gallbladder from liver contour. Exclude the Inferior vena cava when it is discrete and separate from the liver. Include Portal Vein when segment I is on its left. Exclude Portal Vein when segment I is posterior. Include in the contour branches of portal triad [29,69] [α].
Lumbo-sacral Plexus {LumbSacPlex_L LumbSacPlex_R LumbSacPlexs}		At the level of L4–L5 vertebral body: anterior and lateral edges: psoas muscle, common iliac vessels; posterior and medial edges: L4–L5 vertebral body, neural foramina. At the level of S1–S2: anterior and medial edges: psoas muscle; lateral edge: iliacus muscle, sacroiliac joint; posterior edge: sacral ala; medial edge: S1–S2 and neural foramina. Level of superior aspect of piriformis muscle: anterior edge: iliac vessels; posterior edge: piriformis muscle. Level of ischial spine: anterior and medial edges: obturator internus muscle and ischial spine; lateral edge: piriformis muscle; posterior edge: gluteus maximus [72] [δ]. Define *LumbSacPlexs* if the entire lumbo-sacral plexus with bilateral nerve roots is considered. Otherwise, contour *LumbSacPlex_L/R* separately to denote structure laterality [29] [α].
Lung {Lung_L Lung_R Lungs}	CT—lung windows	Limit the contour to the air-inflated lung parenchyma without inclusion of any fluid visible on CT. Include small sized vessels (<1 cm or beyond the hilar region); exclude the proximal bronchial tree. Do not include the trachea/bronchus. Automated contouring tools may be used, with reviewing and editing of the auto-contoured structure often required. Considered *lungs* as sum of structures. Normally, the lung dose limits are referred to DVHs of both lungs, with exclusion of the target volume [29,73] [α].
Mitral valve		It is a bicuspid valve and it is located between the left atrium and the left ventricle of the heart. It is composed by two cusps (or leaflets): an anteromedial leaflet and a posterolateral leaflet. A fibrous ring (anulus) surrounds the structure [62] [γ].
Optic Chiasm {OpticChiasm}	T1-weighted MRI	It is located in the subarachnoid space of the suprasellar cistern. Inferior border: pituitary gland. Posterior border: pituitary stalk. Lateral border: internal carotid artery. It originates anteriorly with the optic nerves and it continues dorsally to the optic tracts [37,39,67] [α].
Optic Nerve {OpticNrv_L OpticNrv_R}	T1-weighted MRI	Contour each optic nerve separately. Consider this structure from the posterior part of the eye to the optic chiasm passing through the optic canal to enter the skull [37,39,81] [α].
Oral Cavity {Cavity_Oral}		Cranial and posterior edge: hard palate. Caudal edge: tubercle of hyoid bone. Anterior and lateral edge: inner surface of mandibular and maxillar bone. Include also: oral tongue, soft palate, uvula, and the base of tongue [29,67] [α].
Ovaries {Ovary_L Ovary_R Ovaries}	T2-weighted MR	Contour each ovary separately. Located in the ovarian fossae, proximate to the lateral pelvic wall. The right ovary is usually medial to ileocaecal junction, caecum, and appendix. The left ovary is adjacent to the sigmoid colon. Posteriorly they face the peritoneum. *Ovaries* may be considered as a sum of the two structures [29,66] [α] [82,83] [γ].
Pancreas {Pancreas}		Located at the level of the L1-L3 vertebral bodies. The pancreatic head can be identified to the right of the superior mesenteric artery. The uncinate process is placed posteriorly to the superior mesenteric vein. The pancreatic body lays between the coeliac trunk and superior mesenteric artery, anterior to the aorta [29,69] [α].
Parotid Gland {Parotid_L Parotid_R Parotids}	CT	Contour each gland separately. Anterior: surface of the masseter muscle; the deep lobe of the parotid gland may extend alongside the medial border of the mandible and the medial pterygoid muscle. Medial: the parapharyngeal space. Lateral: subcutaneous fat and the platysma. Superior: the external auditory canal and mastoid process. Caudal: posterior submandibular space, inferior to the mandibular angle. The external carotid artery, the retromandibular vein, and the extracranial facial nerve are enclosed in the parotid gland. Volume and position of the gland can be variable. Consider *Parotids* a sum of two volumes for dose reporting purposes [29,67] [α].
Penil Bulb {PenileBulb}	T2-weighted MRI, or CT with contrast in the urethra	Consider the portion of the bulbous spongiosum of the penis immediately inferior to the GU diaphragm, limited anteriorly by the urethra. Do not extend into the shaft or pendulous portion of the penis. [29,66] [α]
Pericardium		It is called also pericardial sac; it is a fibro-serous sac containing the heart and the roots of the great vessels. It is composed by an outer layer (fibrous pericardium) and an inner layer (serous pericardium) [62] [γ].
Pharyngeal constrictor muscles {Musc_Constrict}		Include the whole muscle constrictor structure, such as the superior, middle, and inferior pharyngeal constrictor muscle. Cranial edge: pterygoid plates (caudal limit) Caudal edge: arytenoid cartilages (caudal limit). Posterior edge: pre-vertebral muscle. Lateral edge: medial pterygoid muscle (cranially); hyoid bone and thyroid cartilages (caudally). Anterior edge: pterygoid hamulus [29] [α] [75] [β].
Pharyngeal constrictor muscles Inferior {Musc_Constrict_I}		Cranial edge: hyoid bone (caudal limit) Caudal edge: arytenoid cartilages (caudal limit). Posterior edge: pre-vertebral muscle. Lateral edge: thyroid cartilage (superior horn) Anterior edge: thyroid cartilage (posterior border) [29] [α] [75] [β].
Pharyngeal constrictor muscle Middle {Musc_Constrict_M}		It originates at the horns of the hyoid bone. Cranial edge: C3 vertebral body (upper edge) Caudal edge: hyoid bone (caudal limit). Anterior edge: base tongue and hyoid bone. Posterior edge: prevertebral space. Exclude pharyngeal lumen from the volume [29] [α] [75] [β].
Pharyngeal constrictor muscle Superior {Musc_Constrict_S}		Cranial edge: pterygoid plates (caudal limit) Caudal edge: C2 vertebral body (caudal limit). Posterior edge: pre-vertebral muscles. Anterior edge: pterygoid hamulus, pterygoid-mandibular raphe, and base of tongue Exclude pharyngeal lumen from the volume [29] [α] [75] [β].
Pituitary Fossa {Fossa_Pituitary}		It is defined as the inner bony limit of the sella turcica and can be considered as an alternative anatomical structure for the pituitary gland [29,37,67] [α].
Pituitary Gland {Pituitary}	MRI	Located upon the hypophysial fossa of the sphenoid bone. Surrounded by a small bony cavity (sella turcica) covered by a dural fold (diaphragm sellae). Connected to the hypothalamus by its pituitary stalk [29,37] [α]. *Fossa_Pituitary*, can be contoured as an alternative structure [29] [α].
Proximal Brochus {Bronchus Prox}	CT—mediastinal window	Consider the volume from the latest 2 cm of the trachea and the carina. Follow the bronchial tree and include right and left mainstem bronchi, right and left upper lobe bronchi, intermedius bronchus, right middle lobe bronchus, lingular bronchus, and the right and left lower lobe bronchi. End lobar bronchi contour at the level of segmental bifurcation [29,73] [α].
Pulmonary valve		It is also called pulmonic valve, a semilunar valve, located between the right ventricle and pulmonary artery. It is composed by three semilunar cusps—two anterior and one posterior, projecting into the lumen of pulmonary trunk [62] [γ].
Rectum {Rectum}		Cranial: consider sigmoid junction when the rectum loses its round shape in the axial plane and connects with the sigmoid. Caudal: the anorectal junction, at the lowest level of the ischial tuberosity (right or left) [29,66] [α].
Retina {Retina_L Retina_R Retinas}		Contour each structure separately. It is structure located in the posterior wall of the eye. Consider the posterior wall of the eye and contour from the insertion of the lateral rectus muscle to the contralateral medial rectus muscle. *Retinas* can be considered as sum of two volumes [29] [α].
Right Coronary Artery (RCA)		Proximal: from the exit of LMCA, it runs in the atrioventricular groove between right ventricle and right atrium, with caudal limit before it reaches the position near the pericardium. Middle: from the distal part of proximal-RCA, it runs in the atrioventricular groove, to the acute heart border. Distal: from the acute hearth border, continues in the atrioventricular groove posteriorly to the crux cordis. Posterior descending artery: from distal RCA it continues between left and right ventricles [64,80] [α].
Sigmoid Colon {Colon_Sigmoid}		Consider the last part of the colon, caudal to descending colon. Cranial border: where the descending colon turns toward the left to reach the middle line at the level of the third piece of the sacrum. Caudal border: recto-sigmoid junction. Contour the outer boundary of the bowel and includes any bowel contents [29] [α] [62] [γ].
Skin {Skin}		The skin is the 5mm inner rind of the external body contour. Please note actual skin thickness will vary dependent on region of interest [29,37] [α] [62] [γ].
Soft Tissues and Skeletal system		Consider muscle and subcutaneous fat close to target volume area [20] [α].
Spinal Canal {SpinalCanal}	CT—bone windows	Consider the volume according to the inner limits of the spinal canal using bone windows [29] [α]. Divided into cervical, thoracic, and lumbar parts. Cranial edge: cortico-medullary junction at the foramen magnum (at the level of C2) Caudal edge: most caudal slice where the spinal canal is visualized, usually at the level of the L5-S1 [67,73] [α] [62] [γ].
SpinalCord {SpinalCordl}	T1-weighted MRI	It is considered the true spinal cord. Cranial edge: level of the tip of the dens of the C2 vertebra, where it continues with the brainstem. Caudal edge: lumbar cistern approximately at the level of L1 vertebral body in adults [29,67] [α].
Spleen {Spleen}	CT—soft windows	Anterior border: stomach. Posterior-lateral border: left 9th to 11th ribs and diaphragm. Medial border: left kidney. Caudal border: left colic flexure. Exclude the peritoneum surrounding the spleen from the contour [29] [α] [62] [γ].
Stomach {Stomach}	CT—oral contrast recommended	Composed by cardia, fundus, body, and antrum and pylorus. Cardia: caudal to the Gastro-esophageal junction. Fundus: the most cephalad portion of the stomach. Body: central, largest portion of the stomach. Antrum: most distal portion, continue into the pylorus [69] [α].
Submandibular Gland {Glnd_Submand_L Glnd_Submand_R Glnd_Submands}		Cranial: Medial pterygoid muscle, mylohyoid muscle. Caudal: Fatty tissue. Anterior: mylohyoid muscle, hyoglossus muscle. Posterior: Parapharyngeal space, sternocleidomastoid muscle. Lateral: medial pterygoid muscle, mandibular bone, platysma. Medial: mylohyoid muscle, hyoglossus muscle, superior and middle pharyngeal constrictor muscle, and digastric muscle. Contour each gland separately. *Glnd_Submands* can be considered as sum of two volumes for dosimetric purposes [29,67] [α].
Supraglottic larynx {Larynx_SG}		It encompasses the epiglottis, supraglottic adductor muscles, aryepiglottic folds, arytenoid cartilages, and false vocal cords. Cranial edge: tip of the epiglottis. Caudal edge: arytenoid cartilages (cranial limit) Anterior edge: hyoid bone, preepiglottic space, and thyroid cartilage. Posterior edge: The inferior pharyngeal constrictor muscles, pharyngeal lumen, and cricoid cartilage. Medial edge: pharyngeal lumen [29,67] [α] [75] [β].
Superior Vena cava		It is located in the anterior right superior mediastinum and it is formed from the confluence of the left and right brachiocephalic veins. It passes behind the first intercostal space, then it ends the sinus venarum of the right atrium [33] [γ].
Temporal Lobe {Lobe_Temporal_L Lobe_Temporal_R}		Posterior edge: consider an imaginary line dividing cranium into anterior 2/3 and post 1/3. Anterior edge: Cerebrospinal Fluid space posterior to greater wing of sphenoid. Superior edge: Sylvian fissure. Inferior edge: base of the skull/tentorium cerebelli. Lateral edge: temporal bone and Cerebrospinal Fluid located medially. Medial edge: inferiorly CSF surrounding brainstem and tentorium cerebelli, superiorly midbrain and lateral ventricles [29] [α] [62] [γ].
Temporo-mandibular -joint {Temporo_mandibula_joint}		Consider the mandibular head, mandibular fossa of temporal bone, and articular eminence form the temporomandibular joint. Located anteriorly to the tragus of the ear [84] [γ].
Testis {Testis_L Testis_R}		Contour each testis separately. Contour the volume along with the tunica vaginalis and epididymis. Exclude the spermatic cord from the contour [29] [α] [62] [γ].
Thyroid Gland {Glnd_Thyroid}		Composed by two connected lobes and is located below the thyroid cartilage. Well defined limits because It has considerable contrast compared to its surrounding tissues. Cranial edge: piriform sinus, Caudal edge: at level of the C5–7 vertebral bodies. Anterior edge: sternocleidomastoid muscles. Posterior-medial edge: the cervical vessels, cricoid cartilage, and esophagus [29,67] [α].
Trachea {Trachea}	CT—mediastinal windows	Cranial limit: cricoid cartilage (caudal edge) Caudal limit: 2cm cranial to the carina. Include in the volume lumen and trachealis muscle [29,73] [α].
Tricuspid valve		It is also called right atrioventricular valve and it is located at the superior portion of the right ventricle. It is composed by three cusps or leaflets: anterior, posterior, and septal cusps. Each leaflet is connected through chordae tendineae to the papillary muscles of the right ventricle [62] [γ].
Ureters {Ureter_L Ureter_R Ureters}		Defined from the ureteropelvic junction of the kidneys. In the abdomen they are located in the retroperitoneum and they lay anteriorly to the psoas muscle. At the level of the ischial spine, the ureter turns anterior and medial and enter the bladder on the posterior bladder aspect in the trigone (ureterovesical junction). *Ureters* can be considered as volume sum [29] [α] [62] [γ].
Urethra {Urethra}	T2-weighted MRI	It origins from the internal urethral orifice at the bladder neck and continues caudally to the external urethral orifice. Contour all muscle layers and use the sagittal view for a better identification. If a urinary catheter is used, pay attention to the anatomy distortion [29] [α] [62] [γ].
Prostatic Urethra {Urethra_Prostatc}	T2-weighted MRI	It is commonly 3–4 cm in length and passes through the prostate gland. The cranial and caudal borders are defined by the limits of the prostate gland. Contour all muscle layers and use the sagittal view for a better identification [29] [α] [62] [γ].
Vagina {Vagina}		Lower Vagina: from introitus to posterior–inferior border of symphysis. Mid vagina: from posterior–inferior border of symphysis to urethra-vesical junction. Upper vagina: from urethra-vesical junction to cervical orifice [85] [γ].

**Legend:** CSF: cerebrospinal fluid; CT: computed tomography scan; DVH: Dose–volume histogram; MRI: Magnetic resonance imaging. The official nomenclature proposed by the Global Harmonization group [29], where present, is given in *{..}.* The anatomical descriptions were classified by level of reliability in their use for DVC evaluation as follows: α—international guidelines or expert consensus in RT contouring, β—validated anatomical description for RT contouring from single institution, γ—anatomical or radiological descriptions from dedicated books or papers, δ—anatomical definition for RT contouring used in planning studies.

## Supplementary Materials

The following supporting information can be downloaded at: https://www.mdpi.com/article/10.3390/curroncol29100552/s1, Supplementary Table S1: (DVCs for emerging OaRs), Supplementary Table S2: (DVCs for RT of Hodgkin’s lymphoma), Supplementary Table S3: (DVCs for RT of breast cancers), Supplementary Table S4: (DVCs for RT of pediatric tumors), Supplementary Table S5: (DVCs for stereotactic ablative RT of ventricular arrythmia) [6,8,11,13,14,15,16,17,18,20,21,22,23,24,35,40,68,79,99,100,101,102,103,104,105,106,107,108,109,110,111,112,113,114,115,116,117,118,119,120,121,122].
