# Supplementary material for: Dose–Volume Constraints fOr oRganS At risk In Radiotherapy (CORSAIR): An “All-in-One” Multicenter–Multidisciplinary Practical Summary
Source: Curr Oncol. 2022 Sep 27;29(10):7021–50. doi: 10.3390/curroncol29100552 (PMC9600677; doi:10.3390/curroncol29100552)
Supplement: Supplementary file 1 [file curroncol-29-00552-s001.zip › curroncol-1877938-supplementary updated.pdf]

Supplementary Material

Table S1. Specific dose-volume constraints for Emerging OaRs.

Organ	Imaging Technique	Constraints (Conventional Fractionation)* (3)
Aorta		$D_{MAX(1\ cm3)}$ <74 Gy [99] [C]
Bichat Fat Pad		No organ Volume reduction with Dmax 70 Gy [100] [D]
Bone Marrow		BM-V_{10Gy} < 90% [68] [C];<br>BM-V_{20Gy} < 84% [101] [C];<br>BM-D_{MEAN} < 25 Gy (optimal);<br>BM-D_{MEAN} < 30 Gy (mandatory) [102][C];<br>LSS-V_{10Gy} < 85% ;<br>LSS-D_{MEAN} < 28 Gy [102] [C];<br>LSS-V_{15Gy} < 85% [101] [C]
Femur		D_{MAX} <60 Gy mandatory<br>D_{MAX} < 50 Gy optimal<br>[103,104] [C]
Hypothalamus	T1-weighted MRI	D_{MAX} <50 Gy [105] [C]
Temporo-mandibular -joint		$D_{MAX(0.1\ cm3)}$ < 70 Gy [35] [A]
Vagina		D_{2cm3}≤ 145 Gy EQD2 (EBRT +BRT) [106] **[C]**<br>$D_{rectovaginalpoint}$ ≤ 65 Gy EQD2 (EBRT +BRT) [107] **[C]** †

Legend: BM: Bone marrow; BRT brachytherapy; DMax: Maximum Dose; DMean: Mean Dose; EBRT: External Beam Radiation Therapy, EQD2: Equivalent dose in 2-Gy fractions; Gy: Gray; LSS: Lumbosacral Spine; OAR: Organ at Risk; The anatomical descriptions are reported in Table 2. Common abbreviations were used in the tables: V◎ = Volume receiving a dose ≥ ◎ Gy, D◎◎ = dose received by % of the organ volume, D◎ = dose received by γ cm3 (the cubic centimeters) of the organ volume, DMAX = maximum dose received by the organ, DMEAN = mean dose received by the organ. Volumes and doses were expressed as percentage (%) or absolute values (cm3 or Gy, respectively).The letters in square brackets indicate the levels of evidence, classified as follows: [A] International guidelines; [B] literature review on clinical or planning studies; [C] Data from results of clinical or planning studies; [D] expert opinions or used in prospective trials. *considered 180-200 cGy/fraction, as defined by Marks et al (3); †Dose to the recto-vaginal point, defined in the referred text. [106].

Table S2. Specific dose-volume constraints for adult patients treated with curative radiotherapy for Hodgkin Lymphoma.

Organ	Constraints (conventional fractionation) (3)
Aortic valve	D_{MAX} < 25 Gy [11] [A]
Bone Marrow	V_{5Gy}: ALARA;<br>V_{10Gy} <50%;<br>V_{25Gy} <25% [11] [A]
Bowel Small	D_{MAX} < 45Gy;<br>V_{15Gy} < 120 cm^3 [11] [A]
Breast	D_{MEAN} <4 (optimal) ;<br>D_{MEAN} <4-15 Gy (acceptable);<br>V_{4Gy} <10% (optimal);<br>V_{4Gy} : 10%-20% (acceptable);<br>V_{10Gy} <10% (acceptable) [108] [A]
Coronary vessels	Avoid hotspots [11] [A]
Heart	D_{MEAN} <8 Gy (recommended) ;<br>D_{MEAN} <15 Gy (acceptable) [11] [A];
Kidney	D_{MEAN} <8 Gy;<br>V_{10Gy} <30%;<br>V_{20Gy} <15% (recommended) ;<br>V_{20Gy} <25% (acceptable) [11] [A]
Lacrimal Gland	V_{20Gy} <80% [11] [A]
Left ventricle	D_{MEAN} <8 Gy (recommended) ;<br>D_{MEAN} <15 Gy (acceptable) [11] [A];
Liver	D_{MEAN} <15 Gy;<br>V_{20Gy} <30%;<br>V_{30Gy} <20% [11] [A]
Lung	V_{5Gy} < 35% (optimal);<br>V_{5Gy} : 35%-45% (accetable);<br>V_{20Gy} < 20% (optimal);<br>V_{20Gy} : 20%-28% (acceptable);<br>D_{MEAN} <8 Gy (optimal);<br>D_{MEAN} <8-12 Gy (accetable) [108] [A]
Mitral valve	D_{MAX} < 25 Gy [11] [A]
Pancreas	Minimize Volume > 36 Gy [11] [A]
Parotid Gland	Ipsilateral: D_{MEAN} <11 Gy (recommended);<br>D_{MEAN}<24 Gy (acceptable);<br>Controlateral: ALARA[11] [A]
Pericardium	D_{100cm3} < 5 Gy<br>[11] [A]
Pharingeal Constrictors Muscles	D_{MEAN} <25 Gy [11] [A]
Pulmonary valve	D_{MAX} < 30 Gy [11] [A];
Oral Cavity	D_{MEAN} <11 Gy [11] [A]
Spleen	D_{MEAN} <10 Gy;<br>V_{5Gy} ≤ 30%;<br>V_{15Gy} ≤ 20% [11] [A]

Stomach	D_{MAX} < 45Gy [11] [A]
Submandibular Gland	Ipsilateral: D_{MEAN} <11 Gy (recommended);<br>D_{MEAN}<24 Gy (acceptable);<br>Controlateral: ALARA[11] [A]
Thyroid Gland	$V_{25\ Gy}$ < 63.5% ;<br>Minimize V30 [11] [A]
Tricuspid valve	D_{MAX} < 30 Gy [11] [A]

Legend: A_LAD: Left Anterior Descending Artery; ALARA: "as low as reasonably achievable", principle of radiation safety; CxCA: Circumflex Coronary Artery; DMax: Maximum Dose; DMean: Mean Dose; Gy: Gray; LMCA: Left Main Coronary Artery, PDA: Posterior Descending Artery; RCA: Right Coronary Artery. Common abbreviations were used in the tables: V◎ = Volume receiving a dose ≥ ◎ Gy, D◎◎ = dose received by % of the organ volume, D◎ = dose received by γ cm3 (the cubic centimeters) of the organ volume, DMAX = maximum dose received by the organ, DMEAN = mean dose received by the organ. Volumes and doses were ex-pressed as percentage (%) or absolute values (cm3 or Gy, respectively). The anatomical descriptions are described in Table 2. The letters in square brackets indicate the levels of evidence, classified as follows: [A] International guidelines; [B] literature review on clinical or planning studies; [C] Data from results of clinical or planning studies; [D] expert opinions or used in prospective trials. *considered 180-200 cGy/fraction, as defined by Marks et al [3].

Table S3. Specific dose-volume constraints for adult patients treated with curative radiotherapy for Breast Cancer.

**Organ**	**Moderate Hypofractionation (40 Gy/15 fx)**	**Ultra- Hypofractionation (5 Fractions)**
Anterior Descend-ing Artery	DMAX(≤ 20 Gy)<br>[109,110][C]	
Breast	D_{MEAN} ALARA;<br>D_{MAX} < 3% of the prescribed dose<br>[111] [D]<br>V_{5Gy} < 15%<br>[112,113] [D]	Contralateral breast<br>D_{MAX} <1 Gy [13] [D] †
Heart	V_{40Gy} < 3%;<br>V_{18Gy} < 5% [114] [D];<br>V_{10Gy} < 5%;<br>V_{2Gy} < 30%; [14] [D];<br>If DIBH :<br>V_{13Gy} < 10%;<br>V_{13Gy} < 2% (optimal);<br>D_{MEAN} < 3 Gy [115] [D]	V_{3Gy} < 10% [13] [D] †;<br>$V_{1.5Gy}$ < 30%;<br>V_{7Gy} < 5 % [14] [D];
Lung	D_{MEAN} <10 Gy;<br>V_{20Gy} < 10% [114] [D];<br>V_{12Gy} < 15% [14] [D];<br>V_{25Gy} < 5%;<br>V_{17Gy} < 8%;<br>V_{8Gy} < 10%<br>D_{MEAN} < 6.36 Gy<br>Contralateral lung $V_{2.5Gy}$ < 15% [111] [D];<br>If DIBH :<br>Ipsilateral lung :<br>V_{18Gy} <10% (optimal);<br>D_{MEAN} <6 Gy (optimal);<br>Contralateral lung :<br>$V_{2.5Gy}$ <3% (optimal);<br>D_{MEAN} <1 Gy (optimal) [115] [D]	Ipsilateral lung :<br>V_{10Gy} <20% [13] [D] †;<br>V_{8Gy} <15% [14] [D];<br><br>Contralateral lung :<br>V_{5Gy} <10% [13] [D] †

Legend: ALARA: as low as reasonably achievable; DIBH: Deep inspiration breath hold; DMax: Maximum Dose; DMean: Mean Dose; Gy: Gray. Consider the anatomical descriptions reported in Table2. Common abbreviations were used in the tables: V◎ = Volume receiving a dose ≥ ◎ Gy, D◎◎ = dose received by % of the organ volume, D◎ = dose received by γ cm3 (the cubic centimeters) of the organ volume, DMAX = maximum dose received by the organ, DMEAN = mean dose received by the organ. Volumes and doses were ex-pressed as percentage (%) or absolute values (cm3 or Gy, respectively). The letters in square brackets indicate the levels of evidence, classified as follows: [A] International guidelines; [B] literature review on clinical or planning studies; [C] Data from results of clinical or planning studies; [D] expert opinions or used in prospective trials. *anatomical description reported in **Table1;** † only for Partial Breast Irradiation.

Table S4. Specific dose-volume constraints for pediatric patients.

Organ	Covetional Fractionation *(3)	Comments
Brain	DMAX 58-59 Gy [17] [B]	5% risk of brain necrosis for doses > 58-59 Gy; 5 % risk of neurocognitive function for doses > 35.7, 29.1, 22.2, 18.1 Gy if irradiated brain volume is 10, 20, 50, 100 %, respectively
Brainstem	DMAX< 55.8 Gy-RBE<br>V_{55Gy} ≤ 6% [116] [C]	
Cochlea	DMEAN < 35 Gy [40] [B]	
Hearth	DMEAN <15 Gy [20] [A]<br>V_{20Gy} < 0.1 %;<br>V_{5Gy} < 50 % [117] [C]	
Lung	V_{24Gy} < 30% [20] [A]	
Optic pathway	DMAX < 30 Gy (optimal);<br>[15] [D]<br>DMAX < 45 Gy (mandatory);[15,40] [B]	Retinopaty, partial blindness, cataracts.
Orbital Bone	$D_{MAX(0.1\ cm3)}$ < 40 Gy-RBE [118] [C]	To avoid dysmorphic alterations of the orbital bones (especially in the radiotherapy of rhabdomyosarcomas)
Pituitary Gland	DMAX < 60 Gy [15] [D];<br>DMAX < 42 Gy;<br>DMEAN < 25 Gy<br>[40] [B]	
Parotid Gland	DMEAN ≤ 26 Gy<br>DMEAN <40 Gy (mandatory) [18] [B]	
Soft Tissues and Skeletal system	Limit V_{25Gy}-V_{30Gy} [20] [A]	Limit V25-30 Gy to reduce muscular hypoplasia.<br>Avoid substantial dose gradient (> 10-15 Gy) to the spine to limit asymmetric growing (kyphosis or scoliosis). [20] [A]
Spleen	DMEAN < 10 Gy [119] [A] [120] [C]	
Submandibular Gland	DMEAN ≤ 26 Gy [18] [B]	
Rectum	D_{5cm3} < 52 Gy EQD2 [121] [C]	Data obtained in a population of pediatric patients with pelvic tumors undergoing pulsed brachytherapy ± external beam radiotherapy
Thyroid Gland	DMEAN <30 Gy ;<br>DMEAN < 20 Gy (optimal);<br>[16] [B]	The predicted average risk of compensated (subclinical) hypothyroidism was 25% and 44% for thyroid doses of 20 and 30 Gy, respectively.<br>From that analysis, the expected rates of uncompensated hypothyroidism were 7% and 13% for thyroid doses of 20 and 30 Gy, respectively. [16] [B]

Legend: ALARA: as low as reasonably achievable; EQD2: equivalent doses per 2-Gy fractions; DMax: Maximum Dose; DMean: Mean Dose; Gy: Gray; Gy-RBE: gray relative biological equivalents. Common abbreviations were used in the tables: V◎ = Volume receiving a dose ⩾ ◎ Gy, D◎◎ = dose received by % of the organ volume, D◎ = dose received by γ cm3 (the cubic centimeters) of the organ volume, DMAX = maximum dose received by the organ, DMEAN = mean dose received by the organ. Volumes and doses were ex-pressed as percentage (%) or absolute values (cm3 or Gy, respectively). The anatomical descriptions are reported in Table 2. The letters in square brackets indicate the levels of evidence, classified as follows: [A] International guidelines; [B] literature review on clinical or planning studies; [C] Data from results of clinical or planning studies; [D] expert opinions or used in prospective trials. *considered 180-200 cGy/fraction, as defined by Marks et al [3].

Table S5. Specific dose-volume constraints for adult patients treated for stereotactic arrhythmia radioablation (STAR).

Organ	Constraints for single dose hypofractionation
Anterior Descending Artery	$D_{MAX(0.03\ cm3)} \leq 12$ Gy [24] [B]<br>$D_{MAX} \leq 14$ Gy [22] [D]
Aorta	$D_{MAX(0.1\ cm3)} \leq 15.4$ Gy [6] [A];
Bowel Small	$D_{MAX(0.5\ cm3)} < 15.4$ Gy (mandatory);<br>$D_{5cm3} < 11.9$ Gy (optimal) [8] [A]
Bowel Large	$D_{MAX} <18.4$ Gy (mandatory);<br>$D_{20cm3} <14.3$ Gy (optimal) [8] [A]
Chestwall	$D_{MAX(0.1\ cm3)} < 30$ Gy (optimal) [6] [A];<br>$D_{1cm3} < 22$ Gy<br>[8] [A]
Esophagus	$D_{MAX(0.1\ cm3)} \leq 15.4$ Gy [6] [A];<br>$V_{11.9Gy} < 5\ cm^3$;<br>$V_{16Gy} < 0.03\ cm^3$ [8,21] [A]
Hearth- PTV	$D_{MAX(0.1\ cm3)} \leq 22$ Gy [6] [A];<br>$V_{16Gy} < 15\ cm^3$;<br>[8,21] [A] [23] [B]<br>$D_{50\%} \leq 5$ Gy [22] [D]
Implantable Cardioverter Defibrillator (ICD)	$D_{MAX} \leq 0.5$ Gy [22] [D]<br>$D_{MAX}< 2$ Gy [80,122] [A]
Left Circumplex Coronary Artery (CxCA)	$D_{MAX(0.03\ cm3)} \leq 12$ Gy [24] [B]<br>$D_{MAX} \leq 14$ Gy [22] [D]
Left Main Coronary artery (LMCA)	$D_{MAX(0.03\ cm3)} \leq 12$ Gy [24] [B]<br>$D_{MAX} \leq 14$ Gy [22] [D]
Liver	$V_{9.1Gy} < 700\ cm^3$;<br>[6,8,21] [A]
Lung	$V_{20Gy} <15\%$ (mandatory);<br>$D_{MEAN} < 8$ Gy (mandatory);<br>$V_{20Gy} <10\%$ (optimal); [6] [A]<br>$V_{7Gy} < 1500\ cm^3$;<br>$V_{7.4Gy} < 1000\ cm^3$;<br>[8,21] [A]
Proximal Brochus	$D_{MAX(0.1\ cm3)} \leq 20.2$ Gy [6] [A]<br>$V_{10.5Gy} < 4\ cm^3$;<br>[8,21] [A]
Right Coronary Artery(RCA)	$D_{MAX(0.03\ cm3)} \leq 12$ Gy [24] [B]
Skin	$D_{MAX(0.01\ cm3)} <26$ Gy (mandatory);<br>$D_{10cm3} < 23$ Gy (mandatory) [6,8] [A]
SpinalCord	$D_{MAX(0.035\ cm3)} <14$ Gy (mandatory);<br>$D_{MAX(0.035\ cm3)} < 12.4$ Gy (optimal) [6] [A]<br>$V_{7Gy} < 1.2\ cm^3$;

	V_{10Gy} < 0.35 cm^3;<br>[8,21] [A]
Stomach	$D_{MAX(0.1\ cm3)}$ <12.4 Gy (mandatory);<br>D_{10cm3} <11.2 Gy (optimal) [6,8] [A]
Superior Vena cava	$D_{MAX(0.1\ cm3)}$ ≤ 15.4 Gy [6] [A];<br>$D_{50\%}$ ≤ 0.6 Gy [22] [D]
Trachea	D_{MAX}≤ 20.2 Gy;<br>$V_{10.5Gy}$ < 4 cm^3; [8,21] [A]

Legend: CxCA: Left Circumplex Coronary Artery; DMax: Maximum Dose; Gy: Gray; ICD: implantable cardioverter defibrillator; A_LAD: Left Anterior Descending Coronary Artery; LMCA: Left Main Coronary artery; RCA: Right Coronary Artery; PTV: Planning Target Volume. The anatomical descriptions are reported in Table 2. Common abbreviations were used in the tables: V◎ = Volume receiving a dose ≥ ◎ Gy, D◎◎ = dose received by % of the organ volume, D◎ = dose received by γ cm3 (the cubic centimeters) of the organ volume, DMAX = maximum dose received by the organ, DMEAN = mean dose received by the organ. Volumes and doses were ex-pressed as percentage (%) or absolute values (cm3 or Gy, respectively). The letters in square brackets indicate the levels of evidence, classified as follows: [A] International guidelines; [B] literature review on clinical or planning studies; [C] Data from results of clinical or planning studies; [D] expert opinions or used in prospective trials. * Please consider anatomical description reported in **Table1 or 2. Note:** other cardiac substructures (Pulmonary trunk, right and left pulmonary veins, right and left ventricles, right atrium, inferior vena cava, phrenic nerve) may be delineated for dosimetric purposes.
